# *In Vitro* and *In Vivo* Antioxidant Activity of a Water-Soluble Polysaccharide from *Dendrobium denneanum*

**DOI:** 10.3390/molecules16021579

**Published:** 2011-02-14

**Authors:** Aoxue Luo, Zhongfu Ge, Yijun Fan, Aoshuang Luo, Ze Chun, XingJin He

**Affiliations:** 1 Department of Landscape Plants, Chengdu Campus of Sichuan Agriculture University, Chengdu 611130, China; 2 United States Geological Survey, Lake Michigan Ecological Research Station, Porter, IN 46304, USA; 3 Chengdu Institute of Biology, Chinese Academy of Sciences, Chengdu 610041, China; 4 College of Life Sciences, Sichuan University, Chengdu 610064, China

**Keywords:** *Dendrobium denneanum*, polysaccharide, antioxidant activity

## Abstract

The water-soluble crude polysaccharide (DDP) obtained from the aqueous extracts of the stem of *Dendrobium denneanum* through hot water extraction followed by ethanol precipitation, was found to have an average molecular weight (Mw) of about 484.7 kDa. Monosaccharide analysis revealed that DDP was composed of arabinose, xylose, mannose, glucose and galactose in a molar ratio of 1.00:2.66:8.92:34.20:10.16. The investigation of antioxidant activity both *in vitro* and *in vivo* showed that DDP is a potential antioxidant.

## 1. Introduction

*Dendrobium denneanum* (Orchidaceae) is a precious herbal plant highly valued in Traditional Chinese Medicine and archived in the Pharmacopoeia of the People's Republic of China. Sections of the stem from the *Dendrobium* species have been used for the treatment of salivary, ophthalmic disorders, fever and chronic superficial gastritis, or as a tonic for promoting the production of body fluids and for improving the quality of life [1]. As for *Dendrobium* species phytochemicals, much research has been carried out on the low molecular compounds, such as bibenzyl [2], phenanthrene [3], and alkaloids [4]. Based on previous studies, polysaccharides exist widely in numerous plants and are identified as essential biomacromolecules in plant life, playing important roles in cell-cell communication, cell adhesion, and molecular recognition in the immune system [5,6,7,8]. In recent years, some bioactive polysaccharides isolated from natural sources have attracted much attention in the field of biochemistry and pharmacology [9,10]. It has been proven that polysaccharides are major active constituents in *Dendrobium* species. Polysaccharides from some *Dendrobium* species, such as *Dendrobium nobile* Lindl [11,12], *Dendrobium huoshanense* [13] and *Dendrobium chrysotoxum* Lindl [14], have been isolated and reported to have beneficial effects on antioxidation, immunity stimulation and antitumor activities. However, the polysaccharides from *Dendrobium denneanum* have been little reported [15]. Therefore, the purpose of the present investigation was to elucidate the isolation and characterization of water-soluble polysaccharide from the stems of *Dendrobium denneanum*, as well as to evaluate its antioxidant activities *in vitro* and *in vivo*.

## 2. Results and Discussion

### 2.1. Polysaccharide isolation and purification

The total yield rate of crude polysaccharides was 15.3% by the boiling-water extraction method, followed by ethanol precipitation. Because of the presence of some colored materials and residual protein, DDP was a brown water-soluble powder. AB-8 and ADS-7 macroporous resin were used for further purification of the resulting DDP. After application of these two macroporous adsorption resins, protein eventually disappeared in the DDP and the polysaccharide DDP had gray-colored powder appearance. The total sugar content of the polysaccharide was estimated to be 99.7% by the phenol-sulfuric acid method. Previous research has indicated that the molecular weight of polysaccharide was an important factor responsible for biological activities [15]. Determining the molecular weight was therefore the first step for the study of the DDP polysaccharide. The molecular weight (M_w_) of DDP was calculated to be 484,700 Da based on the calibration curve obtained with standard dextrans. 

### 2.2. Monosaccharide composition of DDP

**Figure 1:**
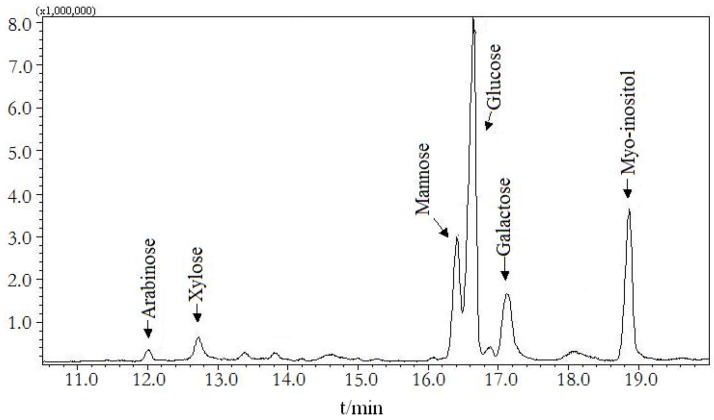
Monosaccharide composition of DDP by GC-MS analysis.

Monosaccharide composition of DDP was analyzed by the trifluoroacetic acid hydrolysis and GC-MS analysis method. The results shown in Figure 1 indicate that glucose is a major monosaccharide constructing the backbones of DDP. DDP was found to be composed of arabinose, xylose, mannose, glucose, and galactose with a molar ratio of 1.00: 2.66: 8.92: 34.20: 10.16.

### 2.3. Infrared spectra of DDP

**Figure 2:**
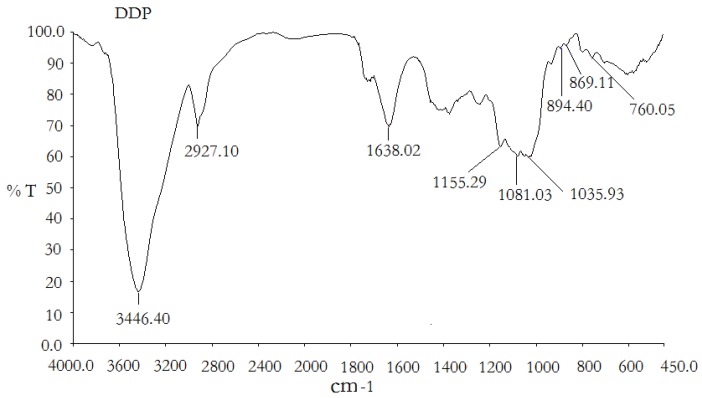
FT-IR spectra of the polysaccharide DDP.

As shown in Figure 2, the sample of DDP exhibited a significant, broad characteristic peak at around 3,446 cm^−1^ for the hydroxyl group, as well as a weak C-H band at around 2,927 cm^−1^ [16]. The band at 1,638 cm^−1^ was due to the bound water [17]. The polysaccharide also appears to have a specific band between 1,200 and 1,000 cm^−1^, which is dominated by ring vibrations overlapped with stretching vibrations of (C-OH) side groups and the (C-O-C) glycosidic band vibration [18]. A characteristic peak at around 894 cm^−1^ was also found in DDP, indicating the β-configuration of the sugar units [19]. Positive specific rotation and characteristic absorption at around 869 cm^−1^ were identified in the IR spectrum, indicating the α-configuration of the sugar units [20].

### 2.4. NMR identification

**Figure 3:**
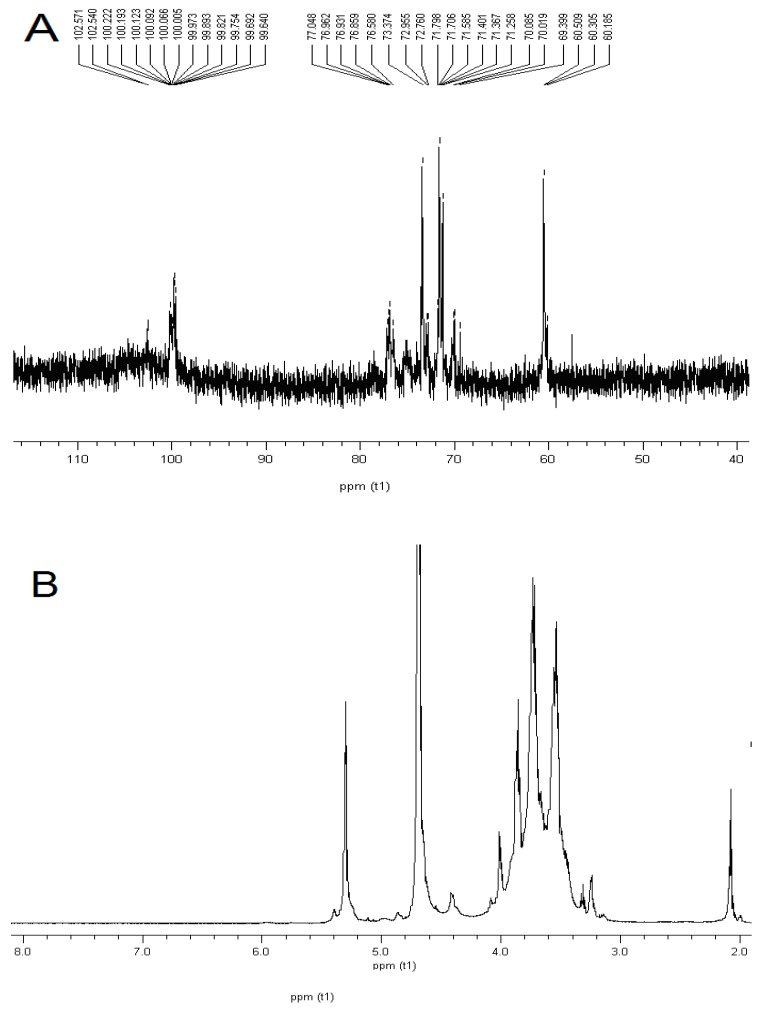
NMR analysis of DDP. A for ^13^C-NMR analysis of DDP, and B for ^1^H-NMR analysis of DDP.

The ^1^H-NMR and ^13^C-NMR results for DDP are shown in Table 1 [21,22,23,24]. Based on the previous discussions in the literature, the signals were centered between δ 99.69 and 102.57 ppm, indicating an α anomeric configuration for all monosaccharide residues of DDP (Figure 3A) [25,26]. The ^1^H-NMR spectrum of DDP showed two anomeric protons at 5.30 and 4.86, which were assigned as (1→4)-α-D-Glcp and (1→6)-α-D-Glcp(Li *et al.* 2008), respectively (Figure 3B).

**Table 1:** ^1^H NMR and ^13^C NMR chemical shifts for DDP in D_2_O at 27 °C.

Sugar residues	H-1	H-2	H-3	H-4	H-5	H-6
	C-1	C-2	C-3	C-4	C-5	C-6
α-D-Galp-(1→	5.12	3.84	3.87	4.01	4.08	3.70
	99.69	69.40	70.02	70.02	71.25	60.51
α-D-Manp-(1→	5.12	4.08	3.87	3.74	3.76	4.00
	102.57	70.02	71.25	69.40	73.37	60.51
→4,6)-α-D-Glcp-(1→	99.69	71.25	73.37	76.58	70.02	65.80
	4.86	3.54	3.70	3.56	4.01	3.66
→4)-α-D-Glcp-(1→	5.40	3.85	3.74	3.63	4.01	3.84
	102.57	73.37	76.58	78.00	76.86	62.50

### 2.5. Antioxidant activities analysis

#### 2.5.1. Effect of scavenging DPPH radicals

**Figure 4:**
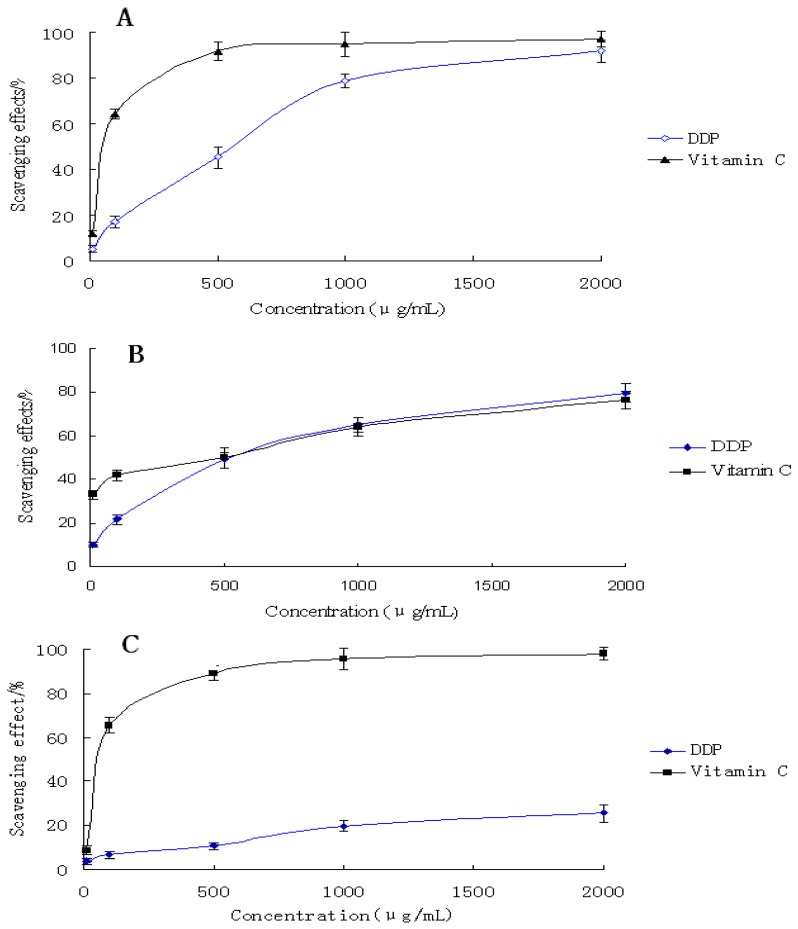
Antioxidant activity of DDP *in vitro*. A: The scavenging effect of DDP on DPPH radical; B: The scavenging effect of DDP on hydroxyl radical; C: The scavenging effect of DDP on ABTS radical. Results are presented as means ± standard deviations.

In this experiment, the scavenging ability of the purified polysaccharide DDP on DPPH free radical were examined in the concentration range of 10–2,000 μg/mL using the DPPH colorimetric assay (shown in Figure 4A

The results indicated that the activities of both samples increased in a concentration dependent manner. Furthermore, the scavenging activities of DDP increased very significantly with increasing concentrations. Especially at the high dose (2,000 μg/mL), DDP exhibited very high radical scavenging activity (92.06%), which was close to that of vitamin C (p < 0.05). However, in the low doses (10 to 1,000 μg/mL), the radical scavenging activity of DDP was lower than that of vitamin C. Therefore, it is obvious that the polysaccharide DDP has strong antioxidant activity in the high doses. 

#### 2.5.2. Scavenging effects of polysaccharide on hydroxyl radicals

The results of hydroxyl radical scavenging activities of DDP compared to those of vitamin C are shown in Figure 4B. The samples exhibited obvious hydroxyl radical scavenging activities in a concentration-dependent manner. The polysaccharide was found to have the ability to scavenge hydroxyl radicals at concentrations between 500 and 2,000 μg/mL. At 500 μg/mL, DDP revealed an excellent hydroxyl radical scavenging activity (48.9%), similar to that of vitamin C (50.1%). At the high dose of 2,000 μg/mL, DDP also exhibited strong activity (79.8%), which was higher than that of vitamin C. These results clearly showed that DDP has potential hydroxyl radical scavenging antioxidant ability.

#### 2.5.3. Scavenging effects of polysaccharide on ABTS

The ABTS radical cation decoloration assay, which employs a specific absorbance (734 nm) at a wavelength well separated from the visible-light range and requires only a short reaction time, has been widely applied to evaluating the total antioxidative activity in both lipophilic and hydrophilic samples [27]. The scavenging ability of DDP on ABTS free radical in the present experiments is shown in Figure 4C. The scavenging power of all samples correlated well with increasing concentrations. Moreover, vitamin C exhibited evident scavenging effect especially at high doses (from 500 to 2,000 μg/mL). At 2,000 μg/mL, the scavenging ability was 98.16%. In contrast, the sample DDP revealed insufficient scavenging power against ABTS radical. Even at a high concentration of 2,000 μg/mL, its scavenging ability was only 25.56%, which was far lower than that of the control. Therefore, the results indicated that the polysaccharide DDP has no significant effect on ABTS radical scavenging.

### 2.6. Antioxidant activity in vivo

**Figure 5:**
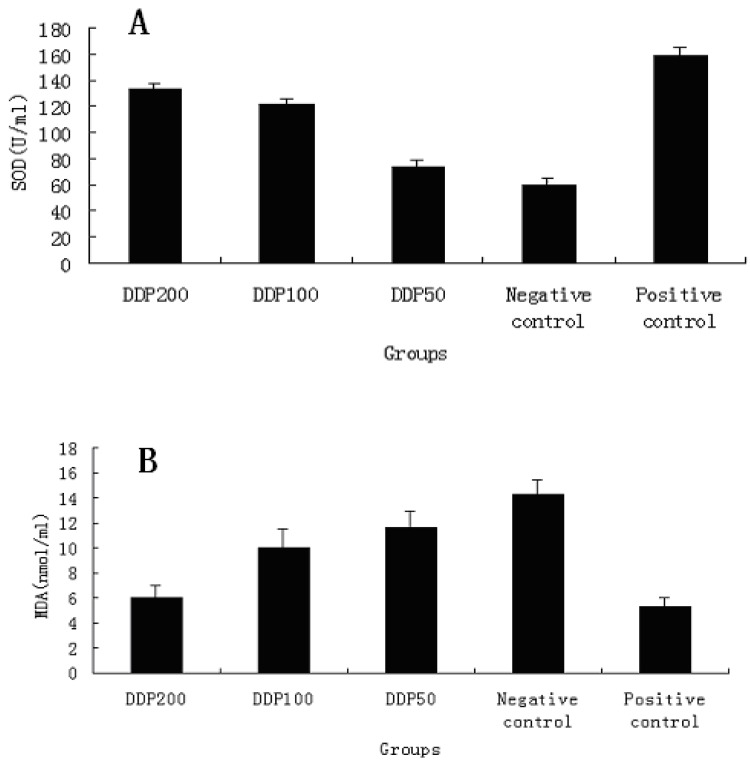
Antioxidant activity of DDP *in vivo*. A: SOD activity analysis in mice; B: Determination of MDA contents in blood serum from the mice. Results are presented as means ± standard deviations.

Superoxide dismutase (SOD) activity (U/mL) was tested with the SOD Assay Kit. Superoxide was generated in xanthine oxidase and hypoxanthine, and the superoxide scavenging effect of serum was determined according to the method of Oyanagui [28]. SOD activity of the serum was expressed in U/mL of the sample. As shown in Figure 5A, SOD activities of different dose of DDP exhibited dose-dependent manner. At 200 mg/kg, particularly, SOD activity of DDP was 132.79 U/mL, which was close to that of vitamin C. However, SOD activity at low concentrations was much less evident, which is similar to that of the negative control. The results were therefore an indication of enhancement SOD activity of DDP for high concentrations. 

The concentrations of MDA in blood serum from the mice were determined with an MDA Assay Kit. The MDA value was estimated according to the thiobarbituric acid (TBA) method [29]. The samples added with TBA were heated in an acidic environment. The absorbance of the resulting solution was measured at 532 nm. The results in Figure 5B exhibit a significant pattern of a decreasing MDA concentration in blood serum with increasing DDP concentration. At 200 mg/kg, the concentration of MDA was 5.95 nmol/mL, close to that of the positive control. This can be interpreted as a significant effect of DDP at high concentrations on MDA scavenging *in vivo*.

## 3. Experimental

### 3.1. Materials and chemicals

DPPH (1,1-diphenyl-2-picrylhydrazyl) radical and Vitamin C were purchased from Sigma (St. Louis, MO, USA). Dextrans of different molecular weights were purchased from Pharmacia Co. (Uppsala, Sweden). The standard monosaccharides (glucose, mannose, rhamnose, galactose, xylose and arabinose) were purchased from the Chinese Institute for the Control of Pharmaceutical and Biological Products (Beijing, China). ABTS (2,2-azinobis-6-(3-ethylbenzothiazoline sulfonic acid) radical was purchased from Merck (Darmstadt, Germany). AB-8 and ADS-7 were purchased from the Chemical Plant of Nankai University (Tianjin, China). SOD Assay Kit001 and MDA Assay Kit A003 were purchased from the Institute of Biological Engineering of Nanjing Jianchen (Nanjing, China). Trifluoroacetic acid (TFA), pyridine, methanol, and acetic acid, ethanol, acetic anhydride and all other chemicals and reagents were analytical grade.

### 3.2. Extraction the crude polysaccharide from Dendrobium denneanum

The stems of *Dendrobium denneanum* were thoroughly washed with water, dried at 60 °C, and then powdered with a pulverizer. The powder was extracted with petroleum ether at 70 °C for 2 h to remove lipids. After filtered, the residues were air-dried, refluxed again with 80% ethanol at 90 °C for 2 h, and then extracted with double-distilled water at 100 °C for 2 h thrice, filtered. The combined filtrate was precipitated with ethanol (4 times the volume of aqueous extract) at 4 °C for 24 h. After filtration and centrifugation, the precipitate was dissolved in double-distilled water and deproteinized five times with the Sevag reagent [30]. It was subsequently dialyzed against deionized water for 72 h and lyophilized, which finally yielded the desired crude *Dendrobium denneanum* polysaccharides (DDP).

### 3.3. Purification of polysaccharide

Two macroporous adsorption resins, AB-8 and ADS-7, were used for purifying the crude polysaccharides of *Dendrobium denneanum*. AB-8 was selected for the decoloration of the DDP. The method was described by Xia *et al.* [31]. ADS-7 was used to remove the residuary protein according to the methods of Li *et al.* [32]. The crude polysaccharides were first dissolved in double-distilled water. After membrane filtration (0.45 μm, Nucleopore), the filtrate was injected to column (26 × 300 mm) of AB-8 and ADS-7, respectively. The columns were eluted with distilled water. The carbohydrate contents of the samples were determined by the phenol-sulfuric acid method using glucose as standard [33]. Protein content was determined according to the method of Bradford *et al.* [34], with bovine serum albumin as standard. 

### 3.4. Determination of molecular weight

The molecular weight of DDP was determined by the Gel Permeation Chromatography (GPC) technique as described by Yamamoto *et al.* [35] using a Waters HPLC apparatus (Waters 515, Waters Co. Ltd., USA) equipped with an Ultrahydrogel Linear Column (300 × 7.8 mm) and a model 2410 Refractive index detector (RID). The column was eluted with 0.2 M phosphate buffer (PH 7.0) with a flow rate of 0.7 mL/min and calibrated with Dextran standards (molecular weights: 2,500, 4,600, 7,100, 10,000, 21,400, 41,100, 84,400, 133,800, 200,000 Da).

### 3.5. Analysis of monosaccharide compositions

DDP (10 mg) was hydrolyzed in a sealed glass tube with 2 M TFA (2 mL) at 100 °C for 8 h [36]. The hydrolysate was dried at a reduced pressure in preparation for acetylation. The acetylation was carried out using hydroxylamine hydrochloride (10 mg) and pyridine (0.5 mL) heated in a water bath at 90 °C for 30 min. The tube was afterwards cooled to room temperature before acetic anhydride (0.5 mL) was added and mixed thoroughly by vortexing. The tube was sealed and incubated in a water bath shaker at 90 °C for 30 min. following a cooling process, an aliquot of the acetylation product (3 μL) was loaded onto an Rtx-5SilMS column (30 m × 0.32 mm × 0.25 μm) of a GC-MS (QP2010, Shimadzu, Japan). This step was carried out under the following conditions: injection temperature: 240 °C; detector temperature: 240 °C; column temperature programmed: 160 °C held for 2 min, increased to 240 °C at a rate of 5 °C/min and finally maintained at 240 °C for 5 min. Nitrogen was used as the carrier gas with a flow rate 1.0 mL/min.

### 3.6. Infrared spectra of DDP

The purified polysaccharide was ground with KBr powder and then pressed into pellets for the estimation of the infrared (IR) spectra of DDP in the 4,000–500 cm^−1^ frequency range. Spectra were recorded on a Perkin-Elmer Fourier Transform IR spectrophotometer (Perkin-Elmer Corp., USA) [37]. 

### 3.7. NMR Identification

Twenty milligrams of DDP was dissolved in D_2_O (0.5 mL, 99.9%), freeze-dried, and redissolved in D_2_O (0.5 mL). The ^1^H-NMR and ^13^C-NMR spectra were measured in an NMR tube (5-mm diameter) at 27 °C with a Bruker Avance 600 spectrometer. The chemical shift was expressed in parts per million (ppm).

### 3.8. Assays for antioxidant activities

#### 3.8.1. DPPH radicals scavenging assay

DPPH radical is a stable free radical that shows a maximum absorption at 517 nm, and is widely used to evaluate the free radical scavenging ability of natural compounds. In the DPPH assay, the antioxidants were able to reduce the stable radical DPPH to the yellow coloured diphenyl-picrylhydrazine. Therefore, the antioxidant activities of a sample can be expressed as its ability in scavenging the DPPH radical. In this study, the antioxidant activities were determined by the DPPH scavenging activities of the purified polysaccharides as described by Shimada *et al.* [38] with some modifications. Vitamin C was used as reference material. Briefly, 0.1-mM solution of DPPH in methanol was prepared, 1.0 mL of which was then mixed with purified polysaccharide solutions (3.0 mL) of various concentrations (10–2,000 μg/mL). The solution was kept at room temperature for 30 min before the absorbance at 517 nm (A_517_) was measured. The DPPH scavenging effect was calculated as follows: DPPH scavenging effect (%) = [A_0_−(A−A_b_)] / A_0_ × 100 (A_0_: the A_517_ of DPPH without the sample; A: the A_517_ of the sample and DPPH; A_b_: the A_517_ of the sample without DPPH). 

#### 3.8.2. Hydroxyl radical scavenging assay

The antioxidant activity was determined by the scavenging effect of hydroxyl radical as described by Smirnoff and Cumbes [39], with some modifications. Briefly, vitamin C was used as reference material. DDP samples of different concentrations (10–2,000 μg/mL) were incubated with 2 mM EDTA-Fe (0.5 mL), 3% H_2_O_2_ (1 mL), and 360 μg/mL crocus in a 4.5-mL sodium phosphate buffer (150 mM, pH 7.4) at 37 °C for 30 min. Hydroxyl radical was detected by monitoring the absorbance at 520 nm. The hydroxyl radical scavenging effect (%) was calculated as [(A_C_−A_S_) / A_C_] × 100, where A_S_ and A_C_ are the A_520_ of the samples and the control group, respectively. The control group used distilled water and a sodium phosphate buffer in place of H_2_O_2_.

#### 3.8.3. ABTS radicals scavenging assay

The ABTS radical cation scavenging activity of DDP was identified using the method given by Re *et al*. [40] with some modifications. ABTS was produced from the reaction of 7 mM of ABTS solution with 2.45 mM of potassium persulphate. The mixture was then kept in the dark at room temperature for 16 h. At the time of use, the ABTS solution was diluted with ethanol to yield an absorbance of 0.70 ± 0.02 at 734 nm. Samples (0.2 mL) of various concentrations (10–2,000 μg/mL) were mixed with ABTS solutions (2 mL). After reaction at room temperature for 6 min, the absorbance at 734 nm was recorded. The ABTS scavenging effect was calculated based on the following formula: ABTS scavenging effect (%) = [A_0_−(As−Ab) ]/A_0_ ×100, where A_0_, As, and A_ b_ are the A_734_ of the ABTS without the sample, of the sample and ABTS, and of the sample without ABTS, respectively. 

#### 3.8.4. Antioxidant activity *in vivo*

Kunming mice (eight weeks old of age, 20 ± 2 g of weight) were provided by Sichuan Academy of Medical Science, China. The mice were kept in separated cages at a temperature of 21 ± 1 °C and a 50–60% of relative humidity. They underwent 12-h light-and-dark cycles with free access to food and water. A total of 50 mice were evenly and randomly divided into five groups, including a D-galactose model control group (negative control), a vitamin C (100 mg/kg) group (positive control), and dose-dependent DDP groups (200, 100, and 50 mg/kg body weight). Each group was induced by a single intraperitoneal injection of D-galactose (100 mg/kg/day body weight, dissolved in a 0.9% saline solution) [41]. The mice in the D-galactose model control group were given a 0.2-mL physiological saline solution (0.9% w/v) once daily for 20 consecutive days by intraperitoneal injection. Twenty-four hours after the last drug administration, blood samples were obtained from the eyepit of the mice and processed for serum. The superoxide dismutase (SOD) activity and the malondialdehyde (MDA) level were also measured.

### 3.9. Statistical analysis

The data were presented as mean ± standard deviation. Statistical analysis was conducted with the SPSS 16.0 software package.

## 4. Conclusions

Polysaccharides in many plants are not only energy resources but they play key biological roles in many life processes as well. The structure and mechanisms of pharmaceutical effects of bioactive polysaccharides on diseases have been extensively studied, and more natural polysaccharides with different curative effects have been tested and even applied in therapies [42]. Recent research has shown that some polysaccharides can play an important role as free radical scavengers for the prevention of oxidative damage in living organisms [43]. In the present study, the water-extracted polysaccharide from *Dendrobium denneanum* was purified with AB-8 and ADS-7 column chromatography, which yielded the polysaccharide DDP. In the subsequent antioxidant experiments *in vitro*, DDP exhibited strong scavenging ability on DPPH radical. The antioxidant effect of DDP is similar to that of vitamin C. The damaging action of OH radical, which can react with all biological molecules such as proteins, lipids, and carbohydrates, is very strong [44] and the resulting oxidative stress can mediate a wide variety of degenerative processes and diseases [45,46]. In the assays above, the DDP polysaccharide exhibited very strong scavenging ability on hydroxyl radical. At the high dose of 2,000 μg/mL, the antioxidant activity of DDP is higher than that of positive control (vitamin C). For *in vivo* assays, the DDP was found to increase the levels of antioxidant enzymes (SOD) and to decrease the MDA content in blood serum. It was confirmed that DDP could protect tissues against oxidative damages. Enhanced SOD activity in mice blood serum also can be related to the *in vivo* antioxidant activity of DDP. With such strong antioxidant ability, DDP was identified as a potential antioxidant.
